# *Chlamydia trachomatis* Infection and Anti-Hsp60 Immunity: The Two Sides of the Coin

**DOI:** 10.1371/journal.ppat.1000552

**Published:** 2009-08-28

**Authors:** Francesco Cappello, Everly Conway de Macario, Valentina Di Felice, Giovanni Zummo, Alberto J. L. Macario

**Affiliations:** 1 Dipartimento di Medicina Sperimentale, Sezione di Anatomia Umana “Emerico Luna”, Università degli Studi di Palermo, Palermo, Italy; 2 Istituto EuroMEditerraneo di Scienza e Tecnologia (IEMEST), Palermo, Italy; 3 Center of Marine Biotechnology, University of Maryland Biotechnology Institute, Baltimore, Maryland, United States of America; The Scripps Research Institute, United States of America

## Abstract

*Chlamydia trachomatis* (CT) infection is one of the most common causes of reproductive tract diseases and infertility. CT-Hsp60 is synthesized during infection and is released in the bloodstream. As a consequence, immune cells will produce anti-CT-Hsp60 antibodies. Hsp60, a ubiquitous and evolutionarily conserved chaperonin, is normally sequestered inside the cell, particularly into mitochondria. However, upon cell stress, as well as during carcinogenesis, the chaperonin becomes exposed on the cell surface (sf-Hsp60) and/or is secreted from cells into the extracellular space and circulation. Reports in the literature on circulating Hsp and anti-Hsp antibodies are in many cases short on details about Hsp60 concentrations, and about the specificity spectra of the antibodies, their titers, and their true, direct, pathogenetic effects. Thus, more studies are still needed to obtain a definitive picture on these matters. Nevertheless, the information already available indicates that the concurrence of persistent CT infection and appearance of sf-Hsp60 can promote an autoimmune aggression towards stressed cells and the development of diseases such as autoimmune arthritis, multiple sclerosis, atherosclerosis, vasculitis, diabetes, and thyroiditis, among others. At the same time, immunocomplexes composed of anti-CT-Hsp60 antibodies and circulating Hsp60 (both CT and human) may form deposits in several anatomical locations, e.g., at the glomerular basal membrane. The opposite side of the coin is that pre-tumor and tumor cells with sf-Hsp60 can be destroyed with participation of the anti-Hsp60 antibody, thus stopping cancer progression before it is even noticed by the patient or physician.

## Hsp60, a Ubiquitous Molecule with Multiple Roles in Health and Disease

Hsp60 is a Group I chaperonin highly conserved during evolution with essential roles in cells and tissues [1]–[4]. In eukaryotes, this chaperonin is usually described as a mitochondrial molecule that works together with its co-chaperonin, Hsp10, to assist in the correct folding of other mitochondrial proteins. The two chaperonins assemble and form an “American football–shaped” molecular complex, a structure that is efficient for correctly folding other proteins, i.e., “client polypeptides” [5]. The Hsp60/Hsp10 complex is typically formed of a double ring-shaped Hsp60 oligomer of 14 monomers and a dome-shaped Hsp10 single ring of seven monomers. Each Hsp60 monomer displays three domains: apical, intermediate, and equatorial [6].

Mammalian Hsp60 has been well characterized [7] and, in humans, its gene resides on Chromosome 2 [8]. Hsp60 proteins are highly conserved in evolution and, therefore, those of eukaryotes and prokaryotes share numerous identical amino acids [2],[7]. This high similarity in primary structure implies common antigenic sites (henceforth called epitopes) that elicit and react with crossreactive antibodies [9],[10]. This is the reason why exogenous Hsp60 from a microbe can elicit an immune response in humans, a response that although directed primarily against the microbial molecule also reacts with the endogenous chaperonin [11], providing a link between infection and development of autoimmune diseases, as postulated for arthritis [12]–[14], multiple sclerosis [15]–[17], and diabetes [9],[18],[19]. These findings have stimulated interest in Hsp60 in physicians willing to understand the molecular basis of disease. However, from the literature it appears that research on circulating Hsp60 and anti-Hsp60 antibodies has been marred by a lack of rigorous quantification of the chaperonin concentrations and antibody titers in plasma or serum. Furthermore, the specificity spectrum of the anti-Hsp60 antibodies has not usually been determined and, therefore, it is difficult if not impossible to know the range of antigens, namely human and bacterial chaperonins, recognized by the antibodies and with what avidity. Because of the two limitations mentioned above, and because of the inherent difficulty in devising reliable strategies to obtain direct correlations between antibody levels and extension of pathological lesions and clinical status in representative samples of pathological specimens and patients and adequate controls, conclusions about the role of anti-Hsp60 antibodies in the onset and progression of disease must be taken as provisory and subject to challenge with further investigation. The main aim of this review is to make pathologists and clinicians fully aware of the existence and pathogenetic potential, which we assume is quite high, of anti-Hsp60 antibodies, but at the same time the review intends to raise awareness on the limitations of previous studies and, thus, encourage new ones more quantitative and accurate in terms of specificity and avidity of the antibodies. In summary, this review presents a field with exciting prospects but full of traps that ought to be recognized within what has been done in the past and what should be avoided in future research.

### Surface and Secreted Hsp60 Can Activate the Immune System

Although Hsp60 is primarily considered a mitochondrial protein, in mammals 20% to 40% of cellular Hsp60 occurs in extra-mitochondrial sites (Table 1) [20]–[23]. The presence of Hsp60 on the cell membrane's surface (sfHsp60) has been noted in normal [24], stressed [25], and tumor cells [26]–[28] and was thought to be associated with membrane transport and signaling [24],[29]. An increase in sfHsp60 levels is considered a danger signal for the immune system in as much as it leads to activation and maturation of dendritic cells and generation of an antitumor T cell response [27],[30]. Hsp60 is a ligand of Toll-like receptor 4, part of the innate immune system, and sfHsp60 expression positively correlates with the triggering of apoptotic phenomena [31]. In addition, the expression of sfHsp60 on the lymphocytic membrane has been associated with spontaneous apoptosis and cell lysis [32],[33]. Therefore, Hsp60 translocation through the plasma membrane should not be considered just as a passive, inconsequential event, but as a key step in the pathogenesis of immune system–mediated disorders (Table 2).

**Table 1:** Hsp60 Locations and Functions.

Location	Function(s)	Reference
Mitochondrion	Protein folding	[5],[6]
Cytosol	Control of signal transduction, apoptosis, senescence, glycolysis	[20]–[23]
Cell membrane	Membrane transport, cell–cell signaling, immune system alerting	[24]–[33]
Intercellular interstitium	Either pro- or anti-inflammatory	[25], [49], [50], [59], [61]–[63]

**Table 2:** Pathologic Conditions in Which Surface Hsp60 Has Been Correlated with Pathogenesis.

Condition	Cell	Role	Reference
Cancer	Tumor	Involved in metastatization to lymph nodes and bones and antitumor immune response activation	[26], [44]–[48]
Atherosclerosis	Endothelial	Confers susceptibility to complement-dependent cell lysis	[50]–[52]
Heart failure	Myocardiocyte	Promotes myocyte apoptosis and pro-inflammatory status in myocardium	[25],[34],[53],[55]
Diabetes	Beta, (insulitis)	Becomes target for T cell–mediated beta-cell destruction	[66]

Hsp60 is also secreted from cells and thus reaches the interstitial fluid and the bloodstream [34]. The levels of Hsp60 in plasma of healthy subjects vary over a wide range from undetectable up to over 1,000 ng/mL; nevertheless, plasma levels in any single individual are rather stable, probably because they are under genetic control [35]. Because of this individual stability of Hsp60 plasma levels when changes appear in any given person, they most probably indicate that something is abnormal. This is one important reason why circulating Hsp60 has recently become a potentially useful marker for clinicians, worth measuring in sera of patients affected by a variety of diseases, as will be discussed later. However, due to the variations in Hsp60 levels among individuals, quantification of circulating chaperonin in populations of patients and controls must be carefully done and repeated to obtain representative samples of data amenable to rigorous statistical analysis that, in turn, will provide a satisfactory basis for assessing correlations of Hsp60 levels with pathology.

### Hsp60 Can Be Elevated in Tumors and Cardiovascular Diseases

Previous studies have revealed that the levels of cytosolic Hsp60 in vivo gradually increase during carcinogenetic steps, from normal tissue to dysplasia to fully developed carcinoma, in various organs: uterine exocervix [36], large bowel [37], and prostate [38]. In contrast, in other malignancies cytosolic Hsp60 was found to decrease during carcinogenesis as compared with normal tissue in tumors of the tongue [39], bladder [40], and airways [41],[42].

sfHsp60 occurs in the cell membrane of certain types of tumors [28], where it is associated with p21ras protein [43] and also with alpha-3-beta-1 integrin, which is involved in the adhesion of metastatic breast cancer cells to lymph nodes and bone tissue [44]. It has also been shown that sfHsp60 plays a role in the metastatization of pancreatic carcinoma [26]. sfHsp60 occurs on the membrane of oral tumor cells and seemingly participates in the mechanism of the tumor cell lysis induced by gammadelta T lymphocytes [45]. Experiments in vitro with a number of tumor cells have shown that photodynamic therapy can induce an increase in Hsp60 [46],[47] and membrane surface localization [48].

In addition to carcinogenesis, Hsp60 has been associated with several other pathologies, for example, with atherosclerosis (ATS), a disease that can be serologically monitored by measuring Hsp60 and anti-Hsp60 antibodies [49]. It has been shown that soluble Hsp60 plays a role in activating vascular and immune cells during ATS development [49], and that the levels of complement-activating anti-Hsp60 antibodies are elevated in ATS-related diseases [50]. Hsp60 has been detected on the surface of stressed endothelial cells [51],[52] and, therefore, these cells become susceptible to complement-dependent lysis by anti-Hsp60 antibodies. In light of these findings, ATS has been proposed as an “autoimmune disease due to an immune reaction against Hsp60” [53]. Nevertheless, for the reasons mentioned earlier about quantification of Hsp60 and anti-Hsp60 antibodies, one has to be aware that the role of Hsp60 in ATS pathogenesis is still under scrutiny. For example, while in teenagers a positive correlation was revealed between early ATS and Hsp60 levels [54], the picture is not clear in older individuals with symptomatic ATS, because in these older patients there are so many other variables that must be considered in relation to ATS that to make reliable correlations between plasma Hsp60 levels and disease is practically impossible.

Recently, it has also been shown that stressed myocardiocytes excrete Hsp60 by the exosomal pathway [34], which may reflect the increase in myocardial levels of Hsp60 that double by the end-stage of heart failure [55]. A chronic injury of progressive heart failure resulted from the localization of Hsp60 in the plasma membrane [25]. Moreover, levels of sfHsp60 were positively correlated with myocardiocyte apoptosis and with the release of the chaperonin into circulation, resulting in the activation of the innate immune system with generation of a pro-inflammatory process in the myocardial interstitium [25].

Since sfHsp60 might be involved in the pathogenesis of ATS, by extension one can infer that this chaperonin is implicated in the pathogenesis of cerebrovascular disorders, such as stroke. In this regard it is noteworthy that Hsp60 levels and distribution are altered in various central nervous system conditions that are not primarily due to the failure of blood circulation, such as Alzheimer, Parkinson, and Huntington diseases, which suggests a participation of the chaperonin in the pathogenesis of these diseases unrelated to vascular pathology [56]. It has been shown that Hsp60 expression in cultured human adult astrocytes is induced by cytokines, i.e., interleukins IL-1β, IL-4, IL-6, and IL-10, and TNF-alpha, which leads to the suggestion that Hsp60 plays an important role also in the pathogenesis of autoimmune diseases of the nervous system, like multiple sclerosis [57].

Anti-Hsp60 antibodies have been found to occur in a number of systemic autoimmune disease–associated vasculitides, such as Takayasu arteritis, polyarteritis nodosa, Wegener granulomatosis, and systemic lupus erythematosus [58]. In all of these conditions, however, for the reasons mentioned earlier, the exact nature (specificity) and role of the anti-Hsp60 antibodies in pathogenesis is still incompletely understood.

### Other Diseases Potentially Related to Hsp60

Hsp60 has also been implicated in the pathogenesis of degenerative joint diseases such as rheumatoid arthritis (RA) [59]. This is an autoimmune disorder with pathogenesis and outcome influenced by the balance between the activities of Th-1 and Th-2 cells. Th-1 activation induces secretion by RA synovial-fluid mononuclear cells of pro-inflammatory cytokines such as IL-1 and TNF-alpha, with consequent cartilage damage, whereas Th-2 activation promotes secretion of IL-4, inhibiting Th-1 activity and diminishing inflammation and cartilage damage [60]. In this respect, it is interesting that mycobacterial Hsp60 activates Th-1 production of IL-1 and TNF-alpha, which suppress cartilage proteoglycan synthesis and contribute to cartilage damage [61]. In contrast, human Hsp60 stimulates Th-2 production of IL-4 and determines a lower release of IL-1 and TNF-alpha by Th-1 cells in comparison to non-human Hsp60-stimulated Th-1 cells [62]. It has been postulated that a humoral response against bacterial chaperonin can elicit a crossreaction against the infected host's Hsp60, thus perpetuating local inflammation and destructive processes in cartilage [59],[63]. All of these data, confirmed also in experimental models of adjuvant arthritis [64], suggest that human, but not bacterial, Hsp60 contributes to suppressing inflammation. Unfortunately, the human chaperonin can also serve as an autoantigen in pathological lesions that may attract antibodies, thus contributing to inflammation and tissue destruction. Such considerations have to be taken into account when thinking of therapeutic uses of Hsp60.

Under physiologic conditions, pancreatic beta-cells show Hsp60 only in mitochondria and secretory granules [65], but in pancreatic islets affected by insulitis the chaperonin migrates towards the cytoplasm as well as to the plasma membrane, in which it can be detected by the immune system that, consequently, mounts an immune response [66]. Since it was realized that Hsp60 is one of the most relevant self-antigens for diabetogenic T cell clones, the chaperonin peptide DiaPep277 has been used to slow down beta-cell damage after the clinical onset of diabetes, both in non-obese diabetic mice and human adults [67],[68]. The first results seem encouraging, but further clinical trials are currently in progress to complete the validation of this therapeutic approach.

It has also been hypothesized that Hsp60 plays a role in the pathogenesis of thyroid and adrenal immune diseases characterized by a proliferation of oncocytes, i.e., intensely eosinophilic cells with granular cytoplasm and a very large number of mitochondria [69]. Lately, the chaperonin has been described as a relevant disease-related autoantigen in autoimmune glomerulonephritis [70], juvenile dermatomyositis [71], and both plaque and guttate psoriasis [72].

## Microbial Hsp60, a Strong and Potentially Harmful Antigen

Numerous infections caused by bacteria, fungi, and mycobacteria can trigger an immune reaction against the microbial Hsp60 with the generation of anti-Hsp60 antibodies [73]. In this regard, it is important to bear in mind that anti-Hsp60 antibodies can also be found in healthy subjects (likely elicited by the chaperonin from microbes in the normal digestive tract flora for instance), representing an early non-specific defense mechanism against pathogens [10]. The sharing of considerable similarity in the primary structure of microbial and host Hsp60 predicts antigenic crossreactivity and development of immune reactions against both proteins [11].

Humoral immune reactions to bacterial Hsp60, such as those from *Chlamydia pneumonia* (*CP*) and *Escherichia coli* have been suggested to be involved in the process of vascular endothelial injury during ATS pathogenesis [74]. It has been stated that the risk of crossreactivity between the microbial chaperonin and the human counterpart on the surface of stressed cells of the arterial endothelium is the cost the organism has to pay for protective immunity against microbial molecules [75]. Infections by *Porphyromonas gingivalis* [76]–[78] and *Helicobacter pylori* [79] have been correlated with a higher risk of development of coronary ATS, due to the high crossreactivity of anti-microbial Hsp60 antibodies with human Hsp60. For similar reasons, *E. coli* Hsp60 has also been implicated in the pathogenesis of autoimmune rheumatic [80] and pancreatic [81] diseases, and Sjogren syndrome [82]. Moreover, an increase in the levels of autoantibodies against endogenous Hsp60 precedes the onset of diabetes in cystic fibrosis patients; hyperimmunization with bacterial Hsp60 caused an increase in anti-Hsp60 autoantibodies that was followed by glucose intolerance [83].

### *Chlamydia trachomatis* Hsp60 Is Abundantly Produced during Persistent Infections

Among pathogens, *Chlamydia trachomatis* (CT) is an intracellular bacterium responsible for a sexually transmitted disease. Approximately 4 million cases of CT infections are estimated to occur annually in the United States, although only about one quarter of those cases are diagnosed and treated [84].

CT includes three human biovars: trachoma (serovars A, B, and Ba or C), urethritis (serovars D-K), and lymphogranuloma venereum (LGV; serovars L1, 2, and 3) [85]. Each CT biovar can cause from mild to severe symptoms. Some infected individuals are asymptomatic, which allows spread from person to person before the infection is detected. In the US in 1990, the direct costs of treating CT infections and its complications were estimated at US$4.2 billion [86], while projected costs for the years 2000s were US$10 billion [87]. These figures clearly demonstrate the magnitude of the health and social problems caused by CT.

CT penetrates into epithelial cells as an elementary body and then converts to a reticulate body, the replicating form of the pathogen. During persistent infections, CT produces a large quantity of Hsp60 (CT-Hsp60) [88], which has been implicated in the pathogenesis of autoimmune disorders such as reactive arthritis [89],[90]. CT-Hsp60 might exert an antiapoptotic effect in nascent tumor cells, which would contribute to female genital tract oncogenesis [91],[92].

There are three CT-Hsp60 isoforms: CT-Hsp60-1, mainly found in the reticulate bodies, and CT-Hsp60-2 and CT-Hsp60-3, which are released extracellularly [93]. Prolonged exposure of the immune system to any of the CT-Hsp60 isoforms leads to immune system activation and antibody formation [94]. CT-Hsp60 is able to stimulate production of pro-inflammatory cytokines in endothelial and smooth-muscle cells and macrophages [95], and it can also promote the activation of specific immune cells via a Toll-like receptor [96].

**Figure 1:**
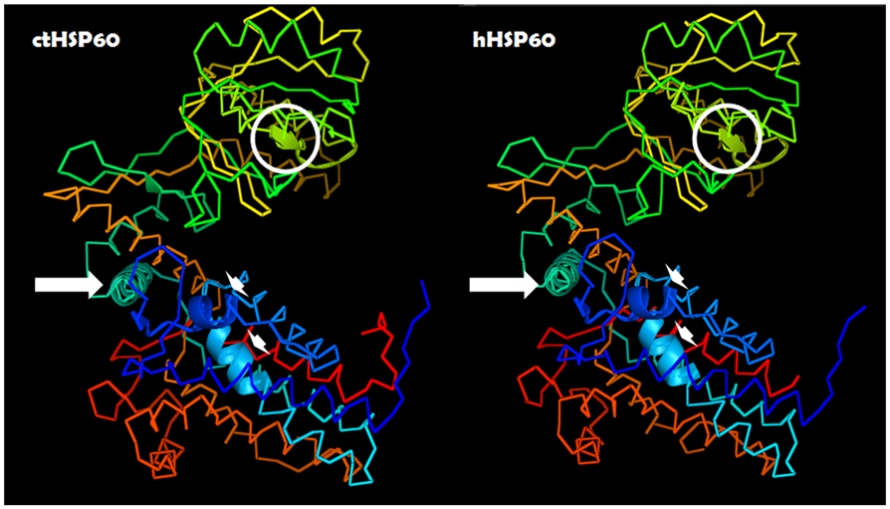
Comparison between the structures of *Chlamydia trachomatis* (ct-) and human- (h-) Hsp60. Shown are the positions of the four epitopes with 100% homologies. Circle: apical domain; arrow: intermediate domain; arrowhead: equatorial domain. See text and reference [94] for further details. The images were created with PyMol (http://pymol.sourceforge.net).

In a recent study, our group compared the amino acid sequences of human-Hsp60 and CT-Hsp60-1 (serovar D), and we found four epitopes with a 100% identity and 13 other peptides of various lengths with identities between 33% and 75% [97]. These epitopes are present in all three domains of the molecule (Figure 1).

Previous comparisons of human versus CT (serovars B, C, and L2) Hsp60 sequences identified 13 major epitopes, seven of which showed crossreactive antibody binding with homologous peptide sequences in human Hsp60 [98]. These data should draw the attention of clinicians towards an often ignored pathogenetic factor, namely, the crossreactive anti-Hsp60 antibodies formed during CT infection. One of the major objectives of this article is to inform clinicians about the occurrence of these antibodies and about their considerable pathogenetic potential.

## *Chlamydia trachomatis* Infection and Antihuman-Hsp60 Antibodies: Negative and Positive Impact

CT infections can persist for very long periods because, usually, the human immune system cannot eliminate pathogens that remain hidden but virulent at focal sites; thus, these silent foci represent a high risk for complications [99].

**Figure 2:**
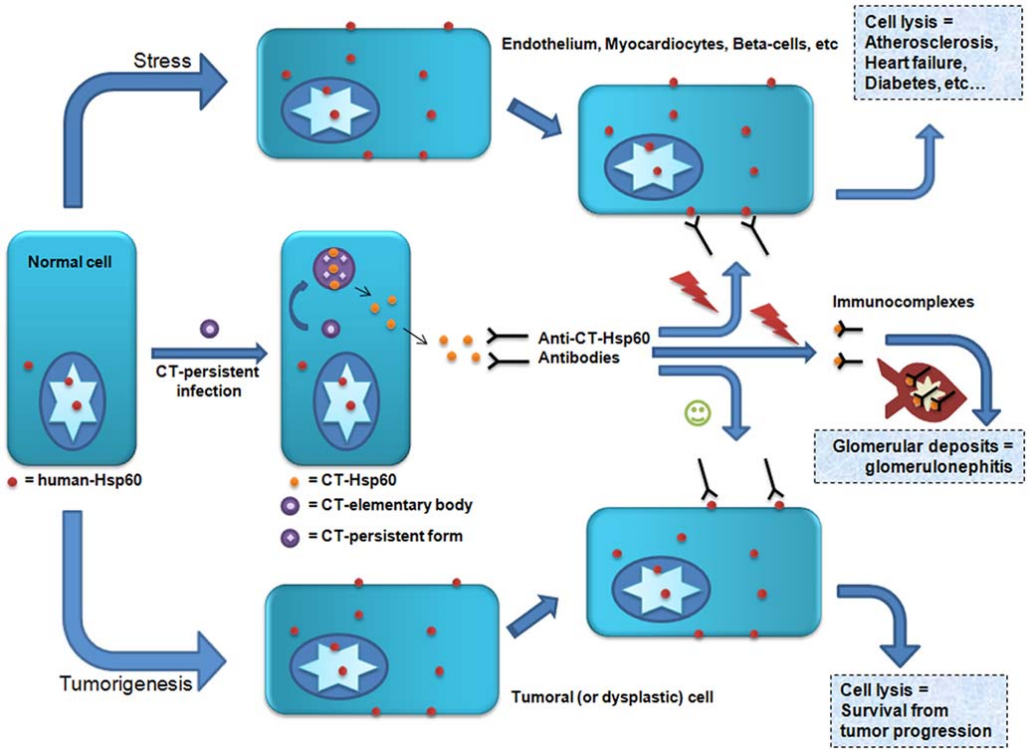
Potential effects of anti-CT-Hsp60 antibodies generated during persistent CT infections. CT-Hsp60 is released from cells infected with *Chlamydia trachomatis* (CT), and anti-CT-Hsp60 antibodies are produced by the host's immune system. In turn, these antibodies recognize surface Hsp60 on either stressed or tumor cells and, consequently, cell lysis and organ destruction can occur, determining pathogenesis of a number of diseases (see text for further details). Likewise, immunocomplexes formed by anti-CT-Hsp60 antibodies and CT- (or human-, not shown) Hsp60 can form deposits in the glomerular basal membrane, causing an idiopathic form of glomerulonephritis. Tumor cell lysis can arrest tumorigenesis, in which case it is likely that the infected individual escapes from cancer without having experienced a detectable pathology or symptom.

People affected by CT develop high titres of serum antibodies anti-CT-Hsp60 [93],[99]. These antibodies also recognize homologous epitopes on human Hsp60 [100], so the more prolonged the infection the greater the increase in the risk that crossreactive antibodies will react against host cells expressing sfHsp60 (Figure 2). These examples illustrate the importance of assessing the specificity spectrum of anti-Hsp60 antibodies as mentioned earlier, to obtain a defined picture of the antibody populations at play in pathology, a consideration that must also be applied when considering CT versus CP infections (see below).

Eukaryotic Hsp60 is a ubiquitous, multifaceted, versatile molecule in as much as it has been classically described in mitochondria, but it has lately been found in extramitochondrial sites [101]. In addition, during carcinogenesis Hsp60 may be augmented or diminished [102]. If augmented, it can have either pro- [103],[104] or anti-apoptotic effects [23],[105] or both [106]. Likewise, when Hsp60 is released outside cells, it can exert either pro- [25],[53] or anti-inflammatory roles [62].

High levels of Hsp60 in plasma of healthy subjects have been positively correlated with low socioeconomic status, social isolation, and psychological distress [107]. Moreover, serum levels of Hsp60, but not those of anti-Hsp60 auto-antibodies, decline with age [108]. These autoantibodies could bind Hsp60 and, thus, produce immunoprecipitates with pathological impact on cells and tissues. There is a report on one case of immune complex glomerulonephritis in a 24-year-old individual with a CT infection of the fallopian tube [109]. Cases like this are probably frequent but missed in clinical practice. Therefore, the occurrence of anti-Hsp60 autoantibodies in individuals with CT infections should always be investigated, paying especial attention to aged patients, since both prevalence of autoimmunity and titres of autoantibodies tend to increase with age [110]–[113].

Anti-Hsp60 autoantibodies recognize Hsp60 epitopes exposed on stressed endothelial cell membranes [51],[52]. This would represent the initial event triggering the formation of ATS lesions and vasculitis. Autoantibodies are also thought to induce apoptosis in cells of the vascular endothelium and to generate renal vasculopathy in systemic lupus erythematosus [114].

When sfHsp60 is exposed on the myocardiocyte plasma membrane, anti-Hsp60 autoantibodies can trigger myocyte destruction via macrophage and/or neutrophil Fc recognition, accelerating heart failure [25]. Studies on Hsp60 autoimmunity also indicate that autoimmune diabetes can begin after bacterial hyperimmunization [83].

### Cautionary Notes on Anti-CT-Hsp60 Antibody Measurements

Although several studies have been carried out to investigate the presence of Hsp60 and anti-Hsp60 in CT-infected individuals (and in various autoimmune diseases, as discussed elsewhere in this article), the results must be subjected to scrutiny and, if possible, to confirmatory research. This is pertinent, for example, to detection of Hsp60 in human plasma.

First, in many of these studies, undiluted plasma was used to measure Hsp60 or antibodies to Hsp60 by ELISA. As a consequence, it becomes very difficult to be certain as to what was actually detected by the antigen–antibody binding assay because undiluted plasma is rich in a wide variety of proteins, which makes nonspecific binding likely. In addition, in a considerable number of cases it is not clear what internal controls were used and how the ELISA was calibrated to avoid false positives and false negatives.

Second, since antibodies to Hsp60 are also found in normal, healthy individuals and since their levels could vary widely (see above [35]), the possible significance of such antibodies, if they are detected in patients, in regard to development of autoimmune diseases can be very difficult to establish.

Third, despite the fact that antibodies to Hsp60 could be induced by a variety of bacteria in human niches, including those present in periodontal pockets and the gastrointestinal tract [10], the antigenic specificity of the antibodies was not, as a rule, determined. Thus, antibodies found to react with CT Hsp60 could very well have been elicited by chaperonins from other microbes, a possibility that must not be ignored due to the known high degree of similarity of Hsp60 across species. Consequently, efforts must be made to obtain and prepare panels of well-characterized antigens to assess the specificity spectrum of the antibodies and identify those most likely to be the relevant ones for any bacterial infection, including CT infection.

Finally, it is of the utmost importance to be aware that CP infections are more widespread in the human population than are CT infections [115]. More than half of all adults in North America have antibodies to CP. As a consequence, one can expect that antibodies to CP Hsp60, which are most likely directed against similar epitopes as those in CT, should be of common occurrence. Here again, the challenge is to determine the specificity spectrum of the antibodies with a panel of Hsp60 molecules from pertinent species and, thus, determine with accuracy the true specificity of the antibodies as well as their titers with regard to the antigen under investigation (e.g., CT or CP Hsp60).

### How, and When, to Defend from CT-Hsp60 Production?

Despite all the suggestive information already in the literature, sfHsp60 has not yet been sufficiently studied in human tissues under normal or pathological conditions, or after stress when Hsp60 translocates to the plasma membrane. sfHsp60 becomes recognizable by the immune system, leading to the generation of autoantibodies and other manifestations of the immune response accompanied by cell destruction, inflammation, and organ damage (Table 3). This series of concatenated events typical of autoimmune disorders due to autoantigens that share sequence homology with human Hsp60 very likely occur in various diseases, for example, Hashimoto disease, thyroiditis, scleroderma, pemphigoid, multiple sclerosis, chronic active hepatitis, primary biliary cirrhosis, and Addison disease [116]. It is imperative to ask the question whether all those diseases, and perhaps others, are not due at least in part to the invasion of the host by microbial Hsp60 from undiagnosed or misdiagnosed (and therefore untreated) bacterial infections, among which CT could be one of the prime suspects. The invading Hsp60 would elicit antibodies primarily directed to the microbial chaperonin but crossreactive with the host's counterpart (Figure 2). If that were the case, an essential distinction should be made between these antibodies elicited by a foreign antigen but reactive also with autoantigens and true autoantibodies elicited by autoantigens. This essential difference could very well mean other important dissimilarities pertaining to structural, functional, and biological molecular aspects, all of which deserve investigation in order to understand pathogenetic mechanisms, and to devise adequate diagnostic and therapeutic strategies.

**Table 3:** Anatomic Sites and Cells in Which Hsp60 Could Play the Role of Autoantigen during Persistent CT Infection and Anti-CT-Hsp60 Antibody Production.

Site	Cell	Pathology	Reference
Vessels	Endothelial cells	Vasculitis, atherosclerosis	[49],[50],[53],[54],[58]
Heart	Myocardiocyte	Myocarditis, infarct, heart failure	[25],[34],[55]
Joints	Synoviocyte	Rheumatoid arthritis	[59], [61]–[63]
Pancreas	Beta-cells	Diabetes	[66],[69]
Thyroid	Thyreocyte	Hashimoto thyroiditis	[69]
Liver	Hepatocyte, biliary duct cells	Chronic active hepatitis, primary biliary cirrhosis	[69]
Adrenal glands	Glomerular zone cells	Addison disease	[69]
Kidney	Glomerulus	Glomerulonephitis	[70]
Skin	Keratinocyte, fibroblast, endothelial cells	Scleroderma, pemphigoid, psoriasis, dermatomyositis	[71],[72]

On the other side of the coin, since sfHsp60 can be present on the surface of tumor cells [26]–[28], the occurrence of an anti-Hsp60 immune response, including circulating autoantibodies, could be beneficial in as much as the response would have a strong negative impact on tumor growth [117]. In this scenario, patients with a chronic CT infection may inadvertently be protected from, or “vaccinated” against as it were, cancer (Figure 2). This is a plausible possibility deserving investigation, even more so because a comprehensive study in vivo on sfHsp60 localization in tumor cells is still lacking.

In regard to the above, in vitro experiments showed that photodynamic therapy could induce sfHsp60 localization in a number of tumor cells [48]. This is one reason why it is believed that the presence of anti-Hsp60 autoantibodies might have antitumor effects and that the use of such antibodies could be a means for cancer treatment.

In summary, the high prevalence of CT infection in humans and the high similarity in the primary structure of CT and human Hsp60 should keep physicians on the alert and drive them to make every possible effort to diagnose CT infection or rule it out. Thus, measuring serum for anti-Hsp60, and also anti-Hsp10, antibodies offers a promising approach if the proper methodology is used (as discussed earlier in various sections of this article). If this diagnostic conduct is abided by, it is likely that much light will be shed on a number of misdiagnosed, idiopathic autoimmune disorders. In addition, investigating autoimmunity elicited by CT Hsp60 could provide information on a suspected protective role of the autoimmune phenomena in cancer, specifically when the cancer cells bear sfHsp60.

## Conclusions and Perspectives

The chaperonin of Group I, Hsp60, or Cpn60 has many important functions, and its alterations, whether genetic or acquired, can cause pathologic disorders [118],[119]. Like other Hsp chaperones, Hsp60 is an evolutionarily conserved protein and, consequently, molecules from different species share sequences that can be antigenic and elicit crossreactive antibodies. This situation is particularly relevant to human diseases with an autoimmune mechanism, particularly in patients with chronic infections.

This article focuses on the potential pathogenetic effect of Hsp60 from *Chlamydia trachomatis* (CT-Hsp60) that shares various antigenic determinants with the human counterpart. Infection with CT leads to an immune response against the invader's chaperonin, but the response crossreacts with the host's Hsp60. The crossreactive effects are perpetuated, and possibly amplified, by the fact that the human chaperonin is present not only inside cells but also outside them, attached to the cell membrane or in circulation. Thus, antibody-antigen reactions can occur on the cell's surface, in the intercellular space, and in biological fluids with a variety of consequences, including modulation of the immune system and generation of pathological lesions with immunoprecipitates. If Hsp60 occurs on the surface of malignant cells, a fact already ascertained for some types of cancer, antibodies or immune cells that react with the chaperonin have the potential ability to damage the tumor.

Since CT infections may go undetected or be misdiagnosed, and may be long lasting, anti-Hsp60 antibodies are likely to be causing disease silently for a long time without the physician being aware of this potentially very damaging situation. Likewise, protracted infections with CT could protect against the growth of certain tumors. These examples demonstrate why Hsp60 is considered a multifaceted, versatile molecule difficult to understand in any particular situation. As a consequence, the physician ought to become aware of the various roles of Hsp60 and anti-Hsp60 antibodies and of the importance of measuring them in all cases with suspected or demonstrated autoimmune manifestations.

It would be important to determine at what stage of human development Hsp60 begins to appear in extracellular locations and whether it is tolerogenic. Elucidation of these aspects of Hsp60 biology is essential to determine if, why, and when anti-Hsp60 autoantibodies (elicited by the endogenous human chaperonin) emerge, and what is their distribution in any given population. Clarification of these points will help understand the mechanism involved in the generation of anti-Hsp60 antibodies and/or their increase in response to invasion by a crossreactive chaperonin from a microbe.

## Accession Numbers

The Entrez Protein Data Bank (http://www.ncbi.nlm.nih.gov/sites/entrez?db=protein) accession codes for the proteins discussed in this paper are *Chlamydia trachomatis* Hsp60-1 (CAH04305, [97]); mitochondrial heat shock 60 kD protein 1 variant 1 [*Homo sapiens*] (ACE06961, [97]); mycobacterial Hsp60 (CAD95638, [120]); *Chlamydia pneumoniae* Hsp60 (AAF38748, [121]); *Escherichia coli* Hsp60 (Q1R3B6, [122]); *Helicobacter pylori* (ACD47477, Reference Kersulyte, D., et al., direct submission, unpublished).
